# Non-Coplanar Diphenyl Fluorene and Weakly Polarized Cyclohexyl Can Effectively Improve the Solubility and Reduce the Dielectric Constant of Poly (Aryl Ether Ketone) Resin

**DOI:** 10.3390/polym15040962

**Published:** 2023-02-15

**Authors:** Feng Bao, Yanxing Liu, Ludi Shi, Jinze Cui, Muwei Ji, Huichao Liu, Jiali Yu, Caizhen Zhu, Jian Xu

**Affiliations:** 1 Institute of Low-Dimensional Materials Genome Initiative, College of Chemistry and Environmental Engineering, Shenzhen University, Shenzhen 518060, China; 2 Chengdu Institute of Organic Chemistry, Chinese Academy of Sciences, Chengdu 610041, China; 3 College of Chemistry and Chemical Engineering, Shantou University, Shantou 515063, China

**Keywords:** poly (aryl ether ketone), permittivity, solubility, cyclohexyl, diphenyl fluorene

## Abstract

With the rapid development of high-frequency communication and large-scale integrated circuits, insulating dielectric materials require a low dielectric constant and dielectric loss. Poly (aryl ether ketone) resins (PAEK) have garnered considerable attention as an intriguing class of engineering thermoplastics possessing excellent chemical and thermal properties. However, the high permittivity of PAEK becomes an obstacle to its application in the field of high-frequency communication and large-scale integrated circuits. Therefore, reducing the dielectric constant and dielectric loss of PAEK while maintaining its excellent performance is critical to expanding the PAEK applications mentioned above. This study synthesized a series of poly (aryl ether ketone) resins that are low dielectric, highly thermally resistant, and soluble, containing cyclohexyl and diphenyl fluorene. The effects of cyclohexyl contents on the properties of a PAEK resin were studied systematically. The results showed that weakly-polarized cyclohexyl could reduce the molecular polarization of PAEK, resulting in low permittivity and high transmittance. The permittivity of PAEK is 2.95–3.26@10GHz, and the transmittance is 65–85%. In addition, the resin has excellent solubility and can be dissolved in NMP, DMF, DMAc, and other solvents at room temperature. Furthermore, cyclohexyl provided PAEK with excellent thermal properties, including a glass transition temperature of 239–245 °C and a 5% thermogravimetric temperature, under a nitrogen atmosphere of 469–534 °C. This makes it a promising candidate for use in high-frequency communications and large-scale integrated circuits.

## 1. Introduction

The rapid development of high-frequency communication and large-scale integrated circuits has raised new requirements for interlayer insulating dielectric materials [1,2,3]. Insulating dielectric materials are required to have a low dielectric constant and low dielectric loss under high frequency, as well as excellent thermal and mechanical properties, processability, dimensional stability, and low water absorption [4,5,6].

Traditional general polymeric materials, such as polyethylene (PE), polypropylene (PP), etc., have low permittivity and dielectric loss but fail to meet requirements due to application–temperature mismatch. Numerous special engineering plastics, such as traditional polyimide (PI) and liquid crystal polymer (LCP), were expected to meet requirements due to their superior thermal and mechanical performance and dielectric properties [7,8]. However, the dielectric constant (D_k_) of 3.0–3.5 is insufficient for application in high-frequency communication and large-scale integrated circuits. Based on the Clausius–Mossotti equation, low-dielectric materials can be prepared by reducing the molecular polarity or increasing the free volume [9]. Considerable research has been conducted on the structural modification of PI and LCP to lower their dielectric constant and meet the requirements for practical applications. Generally, trifluoromethyl-containing substituents [10,11], bulky substituents [12], and alicyclic segments [13] are introduced into the backbone of PI or LCP to reduce polarizability and lower their Dk. Alternatively, the dielectric constant of the resin can be reduced by introducing pore structure through chemical and physical mixing [14]. However, both materials have some limitations in their employment in high-frequency communications and large-scale integrated circuits [15]. PI resin is unstable in hydrothermal environments and has elevated dielectric properties. LCP resin is difficult to process and costly. Therefore, there is an urgent need to design and prepare novel resins with low dielectric constants and dielectric loss, high thermal resistance, and easy processing.

Poly (aryl ether ketone) resins, a high-performance engineering plastic, are widely used in various fields, such as electronics and electrical appliances, aerospace, transportation, and bionic materials, because of their excellent thermal, mechanical, and electrical properties. In addition, traditional poly (aryl ether ketone), such as poly (ether ether ketone) (PEEK), poly (ether ketone) (PEK), etc., have low dielectric constants and dielectric loss over a wide frequency range, and maintain dielectric stability under high humidity and heat exposure conditions [12]. Additionally, their dielectric constant and dielectric loss can be significantly reduced by simple modifications. Therefore, PAEKs have the potential to be used as interlayer dielectric materials in high-frequency communications and large-scale integrated circuits. In sharp contrast, to the best of our knowledge, there is less existing research on the design and preparation of low-dielectric intrinsic poly (aryl ether ketone) resins. Mu et al. reduced the dielectric constant to 1.95~2.21@1MHz by introducing polyhedral oligomeric silsesquioxane (POSS) into the PAEK backbone. Although POSS led to a lower dielectric constant, it came at the expense of thermal properties, and the modified PAEK thermal degraded before 400 °C [16]. Jiang et al. introduced perfluorononenyl pendant groups to obtain a PEEK-PFN-x with a low dielectric constant of 3.0@10KHz, low loss of 0.003@10KHz, and favorable thermal properties [17]. Wang designed and fabricated a poly (arylene ether sulfone) (PES) film containing cyclohexane groups. After foaming, the dielectric constant of the resin was as low as 2.0@10MHz, and the dielectric loss reached 0.005@10MHz [18]. From the above studies, it is not difficult to see that large volume groups, fluorinated substituents, and alicyclic segments can effectively reduce the permittivity of PAEK. Due to their low polarity, alicyclic cyclohexyl groups have been increasingly used to prepare low-dielectric materials [19,20]. However, few studies have reported low-dielectric PAEK resins with cyclohexyl groups.

In addition, conventional PAEKs have regular and symmetric molecular structures with a high degree of crystallinity, presenting insoluble and refractory properties that make processing extremely difficult. This is another obstacle to the application of PAEKs in high-frequency communication and large-scale integrated circuits. Previous studies have shown that polymers with twisted, non-planar molecular junctions often have excellent solubility properties [21,22,23]. This is primarily due to the regularity of the molecular structure being disrupted and the ease with which solvent molecules enter the molecule. Diphenyl fluorene is aromatic, and exhibits stereoscopic distorted conformations [21]. It has been shown that the introduction of diphenyl fluorene into PAEK molecules can lead to materials that are both thermally resistant and soluble [21,24].

To design and prepare novel PAEK resins with low dielectric constants and dielectric loss, high thermal resistance, and easy processing, a difluoride monomer, 1,4-di (4-fluorobenzoyl) cyclohexane (DFBCH), that contains cyclohexyl was prepared to reduce the polarity. A series of novel copolymerized PAEKs containing cyclohexyl and fluorene were prepared by solution polycondensation of DFBCH, 9,9-Bis(4-hydroxyphenyl) fluorene (BHPF), and 1,4-bis(4-fluorobenzoyl) benzene (BFBB). The material’s properties were tuned by controlling the content ratio of the two groups. The effects of the cyclohexyl contents on the properties of the resin were systematically investigated, especially dielectric properties, thermal properties, and solubility properties. It was shown that the introduction of weakly-polarized cyclohexyl groups into the backbone of PAEK effectively improves the solubility, optical transmittance, and dielectric constant of the resin while maintaining its excellent thermal resistance and mechanical properties. This makes it a promising candidate for use in high-frequency communications and large-scale integrated circuits.

## 2. Experimental

### 2.1. Materials

1,4-Cyclohexanedicarboxylic acid (cis-trans isomer mixture) (CHDA, 99%), terephthalic acid (PTA, 99%), thionyl chloride (SOCl_2_, 99%), fluorobenzene (AR), anhydrous aluminum trichloride (AlCl^3^, 99%), N, N-Dimethylformamide (DMF, AR), 9,9-Bis(4-hydroxyphenyl)fluorene (BHPF, 97%), anhydrous potassium carbonate (K_2_CO_3_, AR), sulfolane (99%), and N-Methyl pyrrolidone (NMP, AR) were purchased from Shanghai Maclean Biochemical Technology Co., Ltd. (Shanghai, China). Toluene (AR) was obtained from Xilong Science Co., Ltd., chloroform (CHCl3, AR) was obtained from Shanghai Lingfeng Chemical Reagent Co., Ltd. (Shanghai, China), and concentrated hydrochloric acid(HCl, 37%) was obtained from Guangdong Guangshi Reagent Co., Ltd. (Zhaoqing, China). Acetone was purchased from Sigma-Aldrich Inc. ((Merck, Darmstadt, Germany). All materials were used as received without purification.

### 2.2. Synthesis of DFBCH and BFBB

**Scheme 1:**
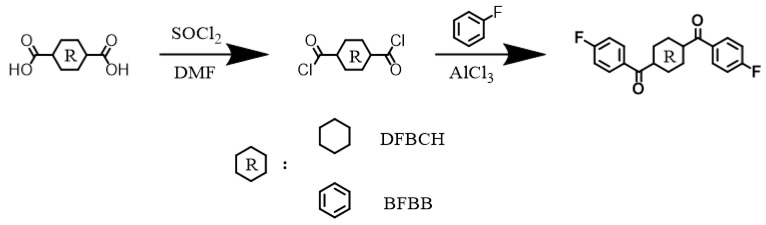
Synthetic route of 1,4-di(4-fluorobenzoyl) cyclohexane (DFBCH) and 1,4-bis(4-fluorobenzoyl) benzene (BFBB).

The synthetic route of DFBCH and BFBB is shown in Scheme 1. A total of 140 g (0.813 mol) CHDA, 280 mL thionyl chloride, and a few drops of DMF were added to a 1 L three-necked flask equipped with a magnetic stirring under nitrogen flow, condenser tube and gas treatment device. The reaction was carried out at 80 °C until the system was transparent, and the excess thionyl chloride was removed by distillation under reduced pressure. The above-synthesized material and 210 g (2.1851 mol) fluorobenzene were added to a 1 L three-necked flask equipped with mechanical stirring under nitrogen flow and spherical condenser ice bath conditions. After the CHDC was fully dissolved, anhydrous aluminum trichloride (214 g, 1.6050 mol) was added, increasing the temperature to 70 °C and maintaining for 12 h. It was then cooled to room temperature and poured into an aqueous solution containing crushed ice, methanol, and hydrochloric acid. The coarse DFBCH was obtained by filtration after vigorous stirring. The white crystalline DFBCH was obtained by double recrystallization with DMF, yield 51% (Supplementary Materials: 1H-NMR, Figure S1; FT-IR spectrum, Figure S2; MS spectrum, Figure S3). The synthesis of BFBB is referred to as the DFBCH synthesis method. The pure BFBB monomer was obtained after two recrystallizations with DMAc, yield 60% (Supplementary Materials: 1H-NMR, Figure S4; FT-IR spectrum, Figure S5; MS spectrum, Figure S6).

### 2.3. Synthesis of PCBEKs

**Scheme 2:**
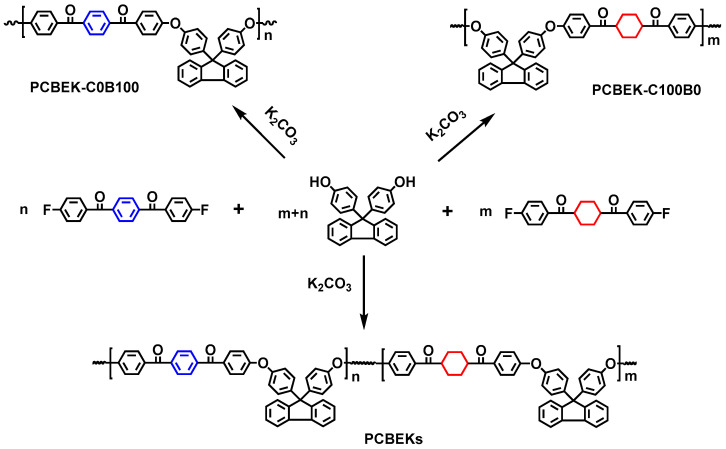
Synthesis of PCBEKs.

Using BHPF, BFBB, and DFBCH as raw materials, poly (aryl ether ketone) resins (PCBEKs) containing cyclohexyl/fluorene structures were synthesized by solution polycondensation with different monomer ratios. The synthetic route is shown in Scheme 2. Resins were named according to the percentage of DFBCH and BFBB. For example, the copolymer resin is named PCBEK-C75B25 when the ratio of DFBCH to BFBB monomeric feedstock is 75:25. In addition, to exclude the influence of the molecular weight of the resin on its macroscopic properties, the molecular weight of the resin was uniformly designed to be 30,000. The preparation of PCBEK-C75B25 is presented below as an example. First, added the BHPF (5.5506 g, 15.84 mmol), DFBCH (4.0093 g, 12.21 mmol), BFBB (1.2764 g, 3.96 mmol), anhydrous potassium carbonate (3.0648 g, 22.176 mmol), 10 mL of mixed solvent (7/1, sulfone/NMP), and 20 mL of toluene into a 100 mL three-necked flask equipped with nitrogen flow, mechanical stirring, a water separation device, and a condensation reflux tube in turn. The reaction system is then heated to a reflux state to promote the BHPF production of phenoxide, and the by-product, water, is brought out with the reflux of toluene. After the water is entirely carried out, the toluene is distilled out and the reaction system is gradually heated to 190 °C. The solvent is added appropriately during the reaction period to suppress locally reactions that are too fast. The reaction is stopped when the viscosity of the reaction system no longer increases. The mixture is then poured into hot water containing hydrochloric acid, and a white fibrous product polymer is obtained, which is the crude product of PCBEK-C75B25.

### 2.4. Preparation of PCBEKs Films

First, the copolymer (1.5 g) was accurately weighed, the solvent NMP (10.5 g) was added, and the ultrasonic treatment was followed by 1 h of agitation at room temperature until the polymer was completely dissolved. The solution was uniformly coated onto a clean, dry glass plate. Last, the glass plate covered with the solution was treated with a series of temperatures (60 °C, 2 h; 800 °C, 1 h; 120 °C, 2 h; 160 °C, 1 h; 200 °C, 2 h) to obtain a PCBEKs copolymer film.

### 2.5. Characterization

The FT-IR spectra of the resins were recorded at room temperature using a Shimadzu IR Affinity-1 spectrophotometer in the 400 to 4000 cm^−1^ range. Bruker AVANCE Ⅲ carried out NMR measurements, and the solvent was deuterated chloroform (CDCl3) or deuterated dimethyl sulfoxide (DMSO-d6). Molecular weight information of the resins was obtained on Agilent PL 200. The thermal properties of the resin were analyzed using TA-Q50 and Netzsch DSC-200F3. The samples were dried at 120 °C for 24 h in a vacuum oven before the test. Dielectric property tests were executed via a vector network analyzer P5004A-200 (Keysight) at 10 GHz room temperature. Solubility tests were performed at room temperature at 0.05 g/mL in different solvents. Wide-angle X-ray diffraction (WAXD) experiments on polymer films were obtained on Rigaku MiniFlex600 using a Cu-Kα radiation (45 kV, 15 mA) source in the range of 5–80° with a scanning rate of 10°/min. The tensile strength of the resin was determined using a SUST CMT5000, according to the ASTM D882-2018 standard. The optical transmittance of the PCBEKs was measured by a UV-3600 Plus (Shimadzu, Japan) at wavelengths from 300 to 800 nm.

## 3. Results and Discussion

### 3.1. Structural Characterization

**Figure 1:**
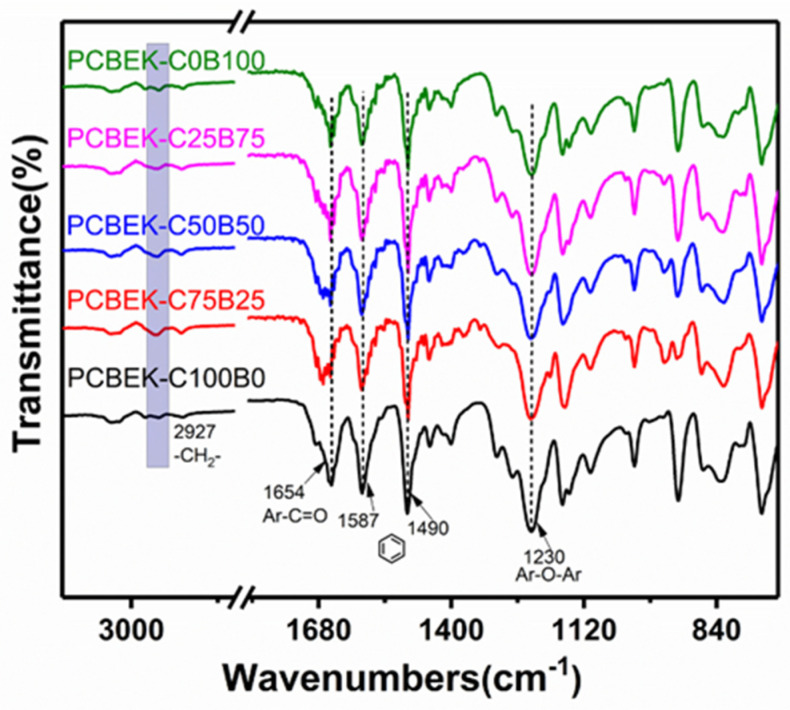
FT-IR spectra of PCBEKs series polymers.

PCBEKs resin films were characterized using ATR-FTIR to determine the functional groups in the molecular chains of the copolymers. As shown in Figure 1, the five characteristic absorption peaks correspond to four distinct functional groups of the copolymer molecular chain. The absorption peak at 1230 cm^−1^ wavenumber is assigned to the C-O-C stretching vibrational of aromatic ether. This indicates that the nucleophilic substitution reaction was successfully performed and formed ether bonds. The absorption peak at 1654 cm^−1^ wavenumber is assigned to the C=O stretching vibrational peak of aromatic ketones. The absorption peaks at 1587 cm^−1^ and 1490 cm^−1^ are characteristic absorption peaks of the benzene ring skeleton. The absorption peak at 2927 cm^−1^ wavenumber is assigned to the stretched vibrational absorption peak of -CH_2_- on the cyclohexane skeleton. Infrared test results preliminarily confirmed that the poly (aryl ether ketone) resin PCBEKs containing cyclohexyl/fluorene structure were successfully prepared.

**Figure 2:**
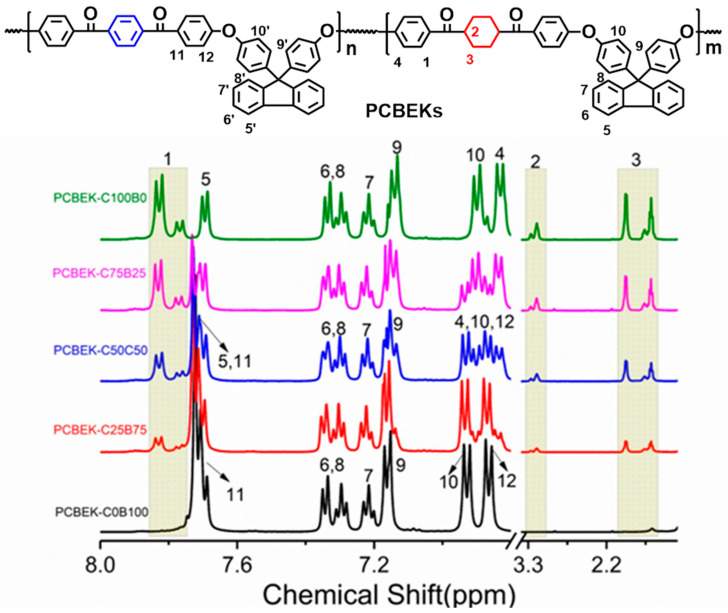
^1^H-NMR spectra of PCBEKs series polymers.

The structure of the copolymer was further analyzed by 1H-NMR, and the results are shown in Figure 2. The signal peaks with δ = 3.98 ppm, δ = 1.94 ppm, and δ = 1.57 ppm are assigned to H2 and H3 of the cyclohexyl structure on DFBCH, respectively. The signal peaks at 7.82 ppm and δ = 7.73 ppm are assigned to H1 and H11. The signal regions H1, H2, and H3 increase with cyclohexanedione content. Based on this, H1 and H11 were selected as the two characteristic protons for comparison. The results showed that the theoretical ratios (1:0, 3:2, 1:2, 2:3, and 0:2) of H1 and H11 were near to the actual ratios (1:0, 3:1.95, 1:1.97, 2:2.08, and 0:2); it can be argued that the actual content ratios of DFBCH and BFBB in repeating units are consistent with the theoretical content ratios. In summary, it can be inferred that the polymer structure is compatible with the design.

It can be seen from the GPC test results (Table 1) that this series of resins have a high molecular weight. The number-average molecular weight (*M_n_*) is 35.6–55.8 KDa, and the weight-average molecular weight (*M_w_*) is 84.2–117.8 KDa. Moreover, the Uhr viscometer test results agree with the GPC test.

### 3.2. Thermal Properties

**Figure 3:**
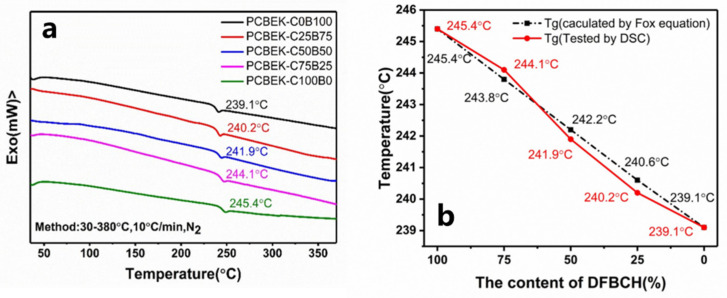
(**a**) DSC curves of PCBEKs series polymers and (**b**) comparison of theoretical values calculated using Fox equation.

Differential scanning calorimeters (DSC) were used to characterize the thermal properties of the copolymer resin. The results are shown in Figure 3 and Table 2. According to the second heating curve of the PCBEK series of resins, only one glass transition process, and no melting process, appears. This indicates that the copolymers were an amorphous structure that matched the XRD results (Figure S7, Supplementary Materials). The glass transition temperature (*Tg*) of resins is between 239.1 °C and 245.4 °C, increasing with the cyclohexanedione content. This is about 100 °C higher than commercial PEEK resin. Presumably, the anti-conformation of the cyclohexanedione monomer improves the molecular chain, which constrains the motion of the polymer at higher glass transition temperatures.

In addition, based on the relationship between the *T_g_* of different monomers and their feed ratio, the *T_g_* value of the copolymer can be predicted by the Fox equation, shown in Formula (1):

(1) $$\frac{1}{T_{g}}=\frac{W_{1}}{T_{g1}}+\frac{W_{2}}{T_{g2}}$$

where *T_g_* is the glass transition temperature of the copolymer, *W*_1_, and *W*_2_ are the mass fractions of a single bisphenol monomer in the total bisphenol, respectively, and *T_g_*_1_ and *T_g_*_2_ are the glass transition temperatures of the corresponding homopolymers. The *T_g_* value of the copolymer measured by DSC and calculated by the Fox equation is shown in Figure 3b. The agreement between experimental and theoretical values of *T_g_* indicates that the structure of copolymers with varying monomer ratios is consistent with predictions.

**Figure 4:**
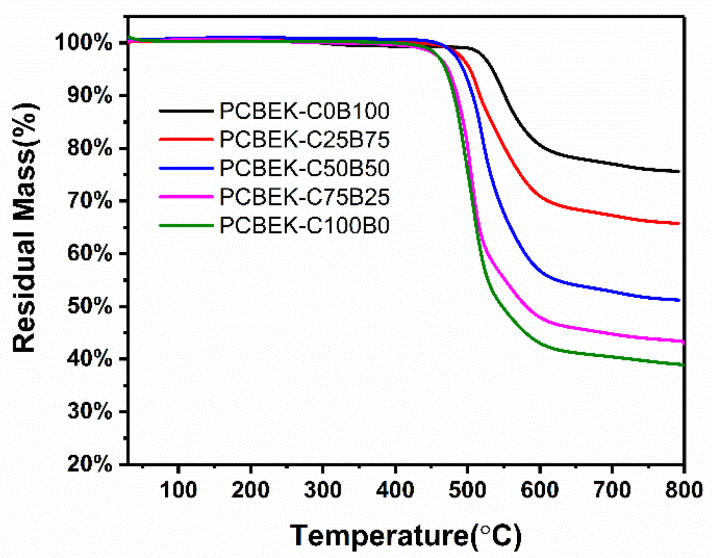
TGA curves of PCBEKs polymers under N_2_ atmosphere.

The thermal stability of the resin was characterized using a thermogravimetric analyzer (TGA), and the results are shown in Figure 4. The PCBEK resins have excellent thermal stability and can exist stably at 450 °C. In the range of 469–534 °C, the resins’ weight can maintain more than 95%. It can be seen from Table 1 and Table 2 that, with the increase of phenyl content, the thermal stability of the resin is increasingly excellent; the *T_d_*_5%_ of PCBEK-C0B100 is 534 °C, and the *T_d_*_10%_ is 551 °C. The carbon residue rate (Cy) at 800 °C is more than 75%. However, the trend of thermal stability of the resins is opposite to the direction of the glass transition temperature trend. The reason is presumed to be that the stability of the alicyclic ring is weaker than that of the benzene ring. The benzene ring structure is steeper and, therefore, more difficult to open than the alkyl rings.

In summary, the thermal performance test results show that the poly (aryl ether ketone) resin containing the cyclohexyl/diphenylfluorene group generally exhibits excellent thermal resistance and thermal stability, and the thermal properties can be adjusted to meet different practical application needs.

### 3.3. Mechanical Properties

**Figure 5:**
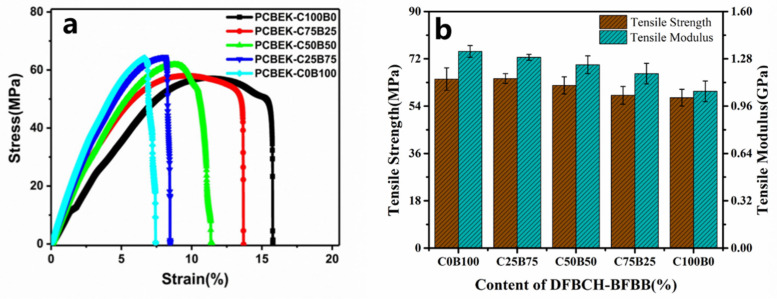
(**a**) The stress-strain curves of the copolymer and (**b**) the tensile strength and tensile modulus of the copolymer.

The resin was made into a film and cut to produce a sample with a particular shape. The tensile stress-strain properties of all samples were measured at room temperature. The tensile strength, Young’s modulus, and elongation of the copolymer at the break values are shown in Figure 5, and the relevant parameters are presented in Table 3. The test results show that the poly (aryl ether ketone) resin containing the cyclohexyl/diphenylfluorene structure has excellent mechanical properties. First, the tensile strength of the PCBEK resin is between 57.2 MPa and 64.5 Mpa, decreasing as cyclohexyl content increases. This may be because the weakly-polarized cyclohexyl group destroys the conjugation effect within the molecular chain, and thus weakens the inter- and intra-molecular interactions. Furthermore, Young’s modulus of the copolymer decreases from 1.33 GPa to 1.05 GPa as the cyclohexyl group in the backbone increases. This may be due to the weaker rigidity of cyclohexyl compared to phenyl. The flexibility of cyclohexyl endows the molecular chains with increased elongation and toughness. In addition, the elongation at the break of the polymer, from 7.45% to 15.71%, increases with the cyclohexyl content, which additionally supports the above analysis. Although the mechanical properties of the modified PAEK resin are reduced, it still meets the requirements of high-frequency communication and large-scale integrated circuits.

### 3.4. Optical Transparency Properties

**Figure 6:**
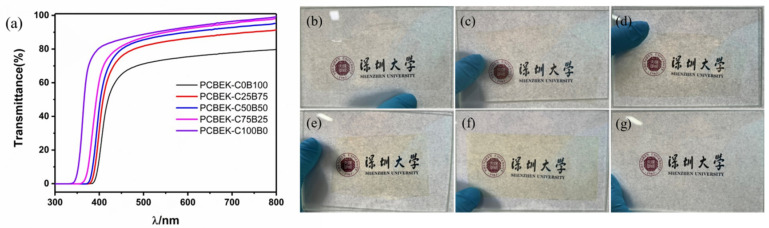
The light transmittance curve of copolymers (**a**) and the digital photographs of PCBEK-C100B0 (**b**), PCBEK-C75B25 (**c**), PCBEK-C50B50 (**d**), PCBEK-C25B75 (**e**), PCBEK-C0B100 (**f**), and blank (**g**).

The optical properties of the film were examined with a UV-Vis-NIR spectrometer. The results of the tests are shown in Figure 6 and Table 4. The cutoff wavelengths of polymer films are in the range of 340 nm to 385 nm. Among these, PCBEK-C100B0 has a cut-off wavelength of 340 nm, and PCBEK-C0B100 has a cut-off wavelength of 385 nm. In addition, polymer films have excellent optical transmittance in the visible light spectrum. The transmittance of PCBEK-C100B0 at 450 nm is 85.81%, which is the best in the series. This is because cyclohexyl units disrupt the conjugation of the molecular chain, which may weaken the effect of electron transfer on the molecular chain [25]. As mentioned above, the resultant copolymers can be widely used as a new optical material in the application of optical thin films.

### 3.5. Solubility Properties

The solubility test results (Table 5) show that PCBEKs can be dissolved in aprotic polar solvents such as NMP, chloroform, DMAc, and DMF at room temperature. In addition, they were partially dissolved in DMSO and sulfolane at room temperature, while soluble after heating. The reason for this is that the kinked and non-coplanar structure of diphenylfluorene breaks the regular arrangement of the molecular chains, enlarging the gaps between the chains and making it easier for the solvent to penetrate, increasing solubility. This excellent solubility makes the copolymer easy to prepare as a thin film by solution pouring.

### 3.6. Dielectric Properties

This study aimed to design a poly (aryl ether ketone) resin with high thermal resistance and low permittivity. The dielectric properties of the resin were characterized using a vector network analyzer, and the results are shown in Table 6. The obtained films show excellent dielectric performance, and the dielectric constant (D_k_) and dielectric loss (D_f_) are 2.95–3.26@10GHz and 0.033–0.041@10GHz, respectively. The dielectric constant of the resin decreases with increasing cyclohexyl content. PCBEK-C100B0 has the best dielectric properties, with a D_k_ of 2.95 and a D_f_ of 0.036 at 10 GHz. Presumably, diphenylfluorene disrupts the regularity of the molecular structure and increases the intermolecular volume. The weakly-polarized cyclohexyl group further reduces the molar polarizability of the molecule. Therefore, the dielectric properties of PCBEK-C100B0 are the best. In addition, low-dielectric materials should be kept as dry as possible during use, as water can significantly impair the permittivity and dielectric loss, and can even damage the device. Wang et al. showed that cyclohexyl could increase the hydrophobicity of resins, which may be another reason for the low dielectric constant of the obtained materials [18]. In conclusion, the introduction of cyclohexyl can effectively reduce the dielectric constant of resin, making it possible for PAEK resins to be used as a candidate for insulating dielectric materials.

## 4. Conclusions

This study synthesized a series of poly (aryl ether ketone) resins that are low dielectric, highly thermally resistant, and soluble, containing cyclohexyl and diphenyl fluorene. The effects of cyclohexyl contents on the properties of a PAEK resin were studied systematically. The results showed that weakly-polarized cyclohexyl groups could reduce the molecular polarization of PAEK, resulting in low permittivity and high transmittance of PAEK, making it possible for PAEK resins to be used as a candidate for insulating dielectric materials. Furthermore, aromatic and non-coplanar diphenyl fluorene can destroy the regularity of PAEK’s molecular chain and provide PAEK with excellent solubility and enriched resin molding processing methods. Herein, it is considered a suitable method for adjusting diphenyl fluorene and cyclohexyl content to acquire low-dielectric insulating materials with comprehensive properties that meet the actual requirements.

## Figures and Tables

**Table 1:** Molecular weight test results of copolymer PCBEKs.

Samples	Composition	GPC ^a^			Viscosity ^b^
	DFBCH/BFBB/BHPF	*Mn* (KDa)	*Mw* (KDa)	PDI	*η* (dL/g)
PCBEK-C100B0	100/0/100	55.8	117.8	2.11	1.39
PCBEK-C75B25	75/25/100	51.3	84.2	1.64	0.96
PCBEK-C50B50	50/50/100	39.7	86.2	2.17	0.68
PCBEK-C25B75	25/75/100	35.6	71.2	2.00	0.77
PCBEK-C0B100	0/100/100	54.1	108.2	2.30	1.32

^a^: Measured by GPC at room temperature with chloroform as mobile phase. ^b^: Measured at room temperature using an Uhr’s viscometer with an initial concentration of 0.5 g dL^−1^.

**Table 2:** Thermal performance parameters of copolymer PCBEKs.

Samples Name	DSC in N_2_	TGA in N_2_		
	*T_g_* (°C) ^a^	*T_d5%_* (°C) ^b^	*T_d10%_* (°C) ^b^	*C_y_* (%) ^c^
PCBEK-C100B0	245.4	469	481	38.9
PCBEK-C75B25	244.1	472	485	43.3
PCBEK-C50B50	241.9	493	507	51.2
PCBEK-C25B75	240.2	503	517	65.7
PCBEK-C0B100	239.1	534	551	75.6

^a^: Measured by DSC under nitrogen atmosphere, temperature range 30–380 °C, 10 °C/min. ^b^: Measured by TGA under nitrogen atmosphere, temperature range 30–800 °C, 20 °C/min. ^c^: Measured by TGA under nitrogen atmosphere, taking the percentage of mass remaining at 800 °C.

**Table 3:** Mechanical property parameters of copolymer PCBEKs films.

Samples Name	Tensile Strength (MPa)	Young’s Modulus (GPa)	Elongation at Break (%)
PCBEK-C100B0	57.2 ± 3.2	1.05 ± 0.14	15.71 ± 0.82
PCBEK-C75B25	58.1 ± 3.3	1.18 ± 0.17	13.70 ± 1.54
PCBEK-C50B50	61.9 ± 3.2	1.24 ± 0.13	11.42 ± 1.02
PCBEK-C25B75	64.5 ± 1.9	1.29 ± 0.04	8.48 ± 1.76
PCBEK-C0B100	64.3 ± 4.2	1.33 ± 0.08	7.45 ± 3.45

**Table 4:** Light transmission performance parameters of copolymer PCBEKs film.

Samples Name	D (µm) ^a^	λ_cut off_ (nm) ^b^	T_450_ (%) ^c^
PCBEK-C100B0	31	340	85.81
PCBEK-C75B25	30	356	81.60
PCBEK-C50B50	37	371	79.84
PCBEK-C25B75	33	379	75.65
PCBEK-C0B100	42	385	64.89

^a^: D is the thickness of the film. ^b^: Cut-off wavelength. ^c^: Transmittance at a wavelength of 450 nm.

**Table 5:** Solubility of copolymer PCBEKs in different organic solvents.

Sample	Solubility ^a^						
	NMP	DMAc	DMF	CHCl_3_	DMSO	Sulfolane	Toluene
PCBEK-C100B0	++	++	++	++	+-	+-	-
PCBEK-C75B25	++	++	++	++	+-	+-	-
PCBEK-C50B50	++	++	++	++	+-	+-	-
PCBEK-C25B75	++	++	++	++	+-	+-	-
PCBEK-C0B100	++	++	++	++	+-	+-	-

^a^: ++, soluble at room temperature; +-, insoluble at room temperature and soluble when heated; -, insoluble when heated.

**Table 6:** Characterization of dielectric constant, dielectric loss, and water absorption of copolymer films.

Samples Name	Dielectric Constant ^a^	Dielectric Loss
PCBEK-C100B0	2.95	0.036
PCBEK-C75B25	3.09	0.041
PCBEK-C50B50	3.11	0.033
PCBEK-C25B75	3.23	0.041
PCBEK-C0B100	3.26	0.036

^a^: Dielectric constant and dielectric loss are measured at 10 GHz with a vector network analyzer.

## Data Availability

Data is available on request from the corresponding author.

## Supplementary Materials

The following supporting information can be downloaded at: https://www.mdpi.com/article/10.3390/polym15040962/s1. Figure S1. 1H-NMR spectrum of DFBCH monomer (500 MHz, Chloroform-d, δ= 8.14 ~ 7.88 (m, 1H), 7.21 ~ 7.04 (m, 1H), 3.45 ~ 3.21 (m, 1H), 2.12 ~ 1.58 (m, 2H). Figure S2. FT-IR spectrum of DFBCH monomer; Figure S3. MS spectrum of DFBCH monomer. Figure S4. 1H-NMR characterization spectrum of BFBB monomer (500 MHz, Chloroform-d): δ= 8.14 ~ 7.88 (m, 1H), 7.21 ~ 7.04 (m, 1H), 3.45 ~ 3.21 (m, 1H), 2.12 ~ 1.58 (m, 2H). Figure S5. FT-IR spectrum of BFBB monomer. Figure S6. MS spectrum of BFBB monomer. Figure S7. WAXD spectra of copolymer PCBEKs.
