# Supplementary material for: Non-Coplanar Diphenyl Fluorene and Weakly Polarized Cyclohexyl Can Effectively Improve the Solubility and Reduce the Dielectric Constant of Poly (Aryl Ether Ketone) Resin
Source: Polymers (Basel). 2023 Feb 15;15(4):962. doi: 10.3390/polym15040962 (PMC9965749; doi:10.3390/polym15040962)
Supplement: Supplementary file 1 [file polymers-15-00962-s001.zip › polymers-2161113-supplementary.pdf]

polymers

MDPI

Supplementary data for

Non-coplanar diphenyl fluorene and weakly polarized cyclo-hexyl can effectively improve the transmittance and reduce the dielectric constant of poly (aryl ether ketone) resin

Bao Feng [1,†,*], Liu Yanxing [1,†], Shi Ludi [2], Cui Jingze [1], Ji Muwei [3], Liu Huichao [1], Yu Jiali [1], Zhu Caizheng [2,*] and Xu Jian [1]

1 Institute of Low-Dimensional Materials Genome Initiative, College of Chemistry and Environmental Engineering, Shenzhen University, Shenzhen, 518060, China

2 Chengdu Institute of Organic Chemistry, Chinese Academy of Sciences, Chengdu, 610041, P.R. China

3 Department of Chemistry, College of Science, Shantou University, Shantou 515063, PR China

* Correspondence: Bao F(bfisvip@163.com;18340878839); Zhu CZ (czzhu@szu.edu.cn)

† These authors contributed equally to this work

This supporting information is composed of the total of 4 pages, including 7 Figures

Page 2-4:

Figure S1. 1H-NMR spectrum of DFBCH monomer (500 MHz, Chloroform-d, δ= 8.14 ~ 7.88 (m, 1H), 7.21 ~ 7.04 (m, 1H), 3.45 ~ 3.21 (m, 1H), 2.12 ~ 1.58 (m, 2H).

Figure S2. FT-IR spectrum of DFBCH monomer

Figure S3. MS spectrum of DFBCH monomer.

Figure S4. 1H-NMR characterization spectrum of BFBB monomer (500 MHz, Chloroform-d): δ= 8.14 ~ 7.88 (m, 1H), 7.21 ~ 7.04 (m, 1H), 3.45 ~ 3.21 (m, 1H), 2.12 ~ 1.58 (m, 2H).

Figure S5. FT-IR spectrum of BFBB monomer.

Figure S6. MS spectrum of BFBB monomer.

Figure S7. WAXD spectra of copolymer PCBEKs.

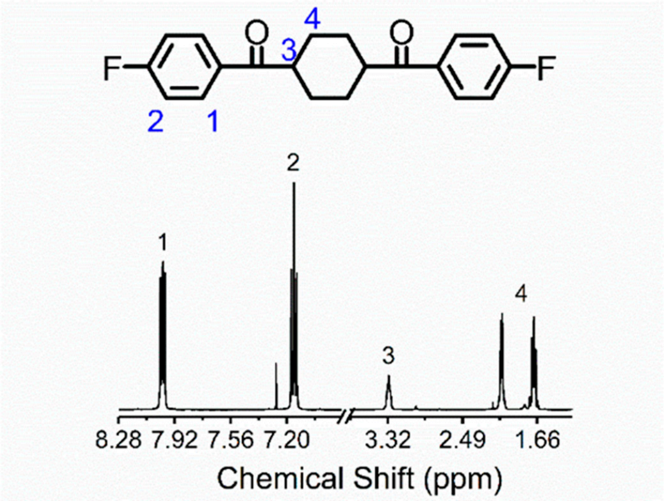

Figure S1. 1H-NMR spectrum of DFBCH monomer (500 MHz, Chloroform-d, δ= 8.14 ~ 7.88 (m, 1H), 7.21 ~ 7.04 (m, 1H), 3.45 ~ 3.21 (m, 1H), 2.12 ~ 1.58 (m, 2H).

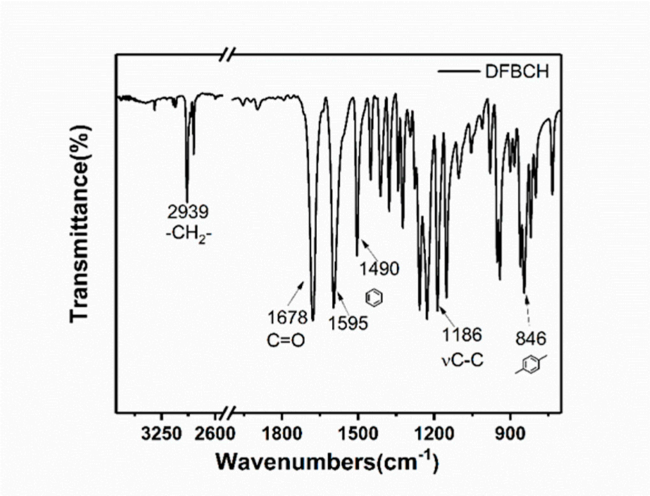

Figure S2. FT-IR spectrum of DFBCH monomer

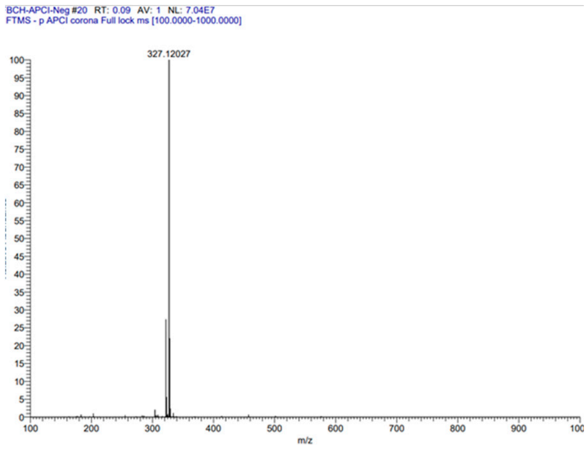

Figure S3. MS spectrum of DFBCH monomer.

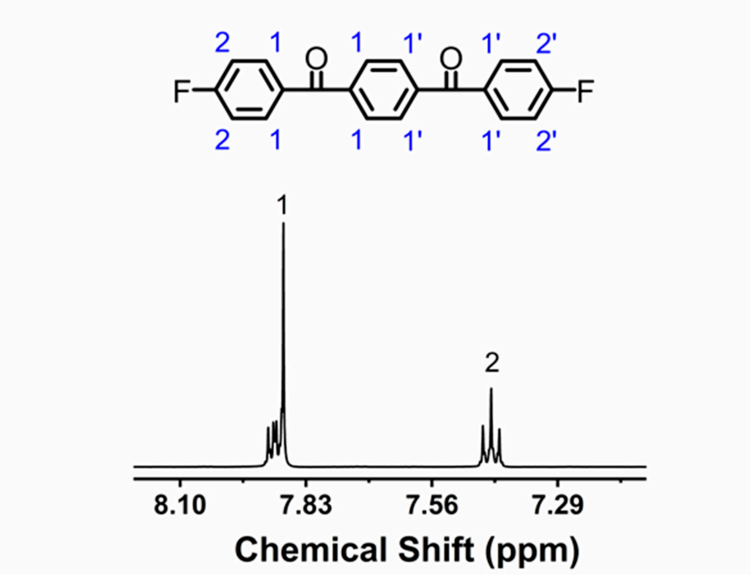

Figure S4. 1H-NMR characterization spectrum of BFBB monomer (500 MHz, Chloroform-d): δ= 8.14 ~ 7.88 (m, 1H), 7.21 ~ 7.04 (m, 1H), 3.45 ~ 3.21 (m, 1H), 2.12 ~ 1.58 (m, 2H).

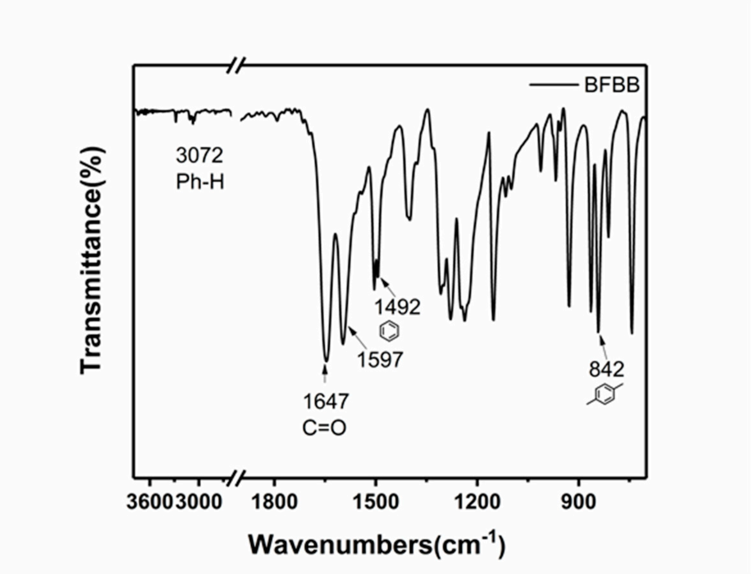

Figure S5. FT-IR spectrum of BFBB monomer.

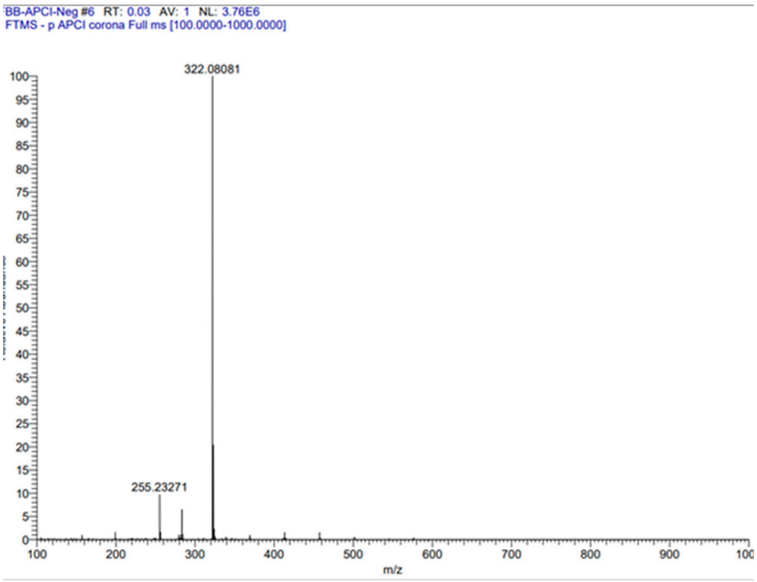

Figure S6. MS spectrum of BFBB monomer.

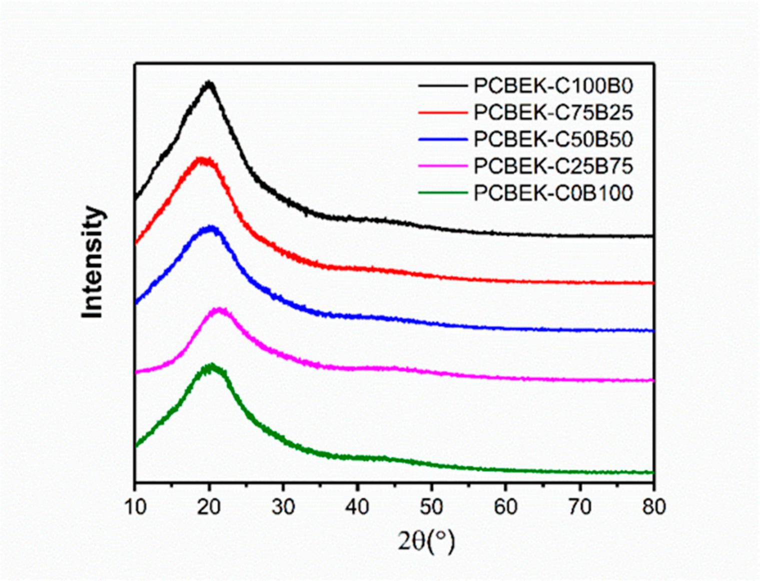

Figure S7. WAXD spectra of copolymer PCBEKs
